# Growth hormone/insulin-like growth factor I axis in health and disease states: an update on the role of intra-portal insulin

**DOI:** 10.3389/fendo.2024.1456195

**Published:** 2024-11-21

**Authors:** Kevin C. J. Yuen, Rikke Hjortebjerg, Ashok Ainkaran Ganeshalingam, David R. Clemmons, Jan Frystyk

**Affiliations:** ^1^ Department of Neuroendocrinology and Neurosurgery, Barrow Neurological Institute, University of Arizona College of Medicine and Creighton School of Medicine, Phoenix, AZ, United States; ^2^ Department of Endocrinology, Odense University Hospital, Odense, Denmark; ^3^ Department of Clinical Medicine, Faculty of Health Sciences, University of Southern Denmark, Odense, Denmark; ^4^ Steno Diabetes Center Odense, Odense University Hospital, Odense, Denmark; ^5^ Department of Medicine, University of North Carolina School of Medicine, Chapel Hill, NC, United States

**Keywords:** growth hormone, insulin-like growth factor-I, intra-portal insulin, acromegaly, adult growth hormone deficiency, hepatic growth hormone sensitivity

## Abstract

Growth hormone (GH) is the key regulator of insulin-like growth factor I (IGF-I) generation in healthy states. However, portal insulin delivery is also an essential co-player in the regulation of the GH/IGF-I axis by affecting and regulating hepatic GH receptor synthesis, and subsequently altering hepatic GH sensitivity and IGF-I generation. Disease states of GH excess (e.g., acromegaly) and GH deficiency (e.g., congenital isolated GH deficiency) are characterized by increased and decreased GH, IGF-I and insulin levels, respectively, where the GH/IGF-I relationship is reflected by a “primary association”. When intra-portal insulin levels are increased (e.g., obesity, Cushing’s syndrome, or due to treatment with glucocorticoids and glucagon-like peptide 1 receptor agonists) or decreased (e.g., malnutrition, anorexia nervosa and type 1 diabetes mellitus), these changes secondarily alter hepatic GH sensitivity resulting in a “secondary association” with discordant GH and IGF-I levels (e.g., high GH/low IGF-I levels or low GH/high IGF-I levels, respectively). Additionally, intra-portal insulin regulates hepatic secretion of IGFBP-1, an inhibitor of IGF-I action. Through its effects on IGFBP-1 and subsequently free IGF-I, intra-portal insulin exerts its effects to influence endogenous GH secretion via the negative feedback loop. Therefore, it is important to understand the effects of changes in intra-portal insulin when interpreting the GH/IGF-I axis in disease states. This review summarizes our current understanding of how changes in intra-portal insulin delivery to the liver in health, disease states and drug therapy use and misuse that leads to alterations in GH/IGF-I secretion that may dictate management decisions in afflicted patients.

## Introduction

The growth hormone (GH)-insulin-like growth factor I (IGF-I) axis plays a critical role in promoting linear growth in children (1), whereas in adults, its role is primarily of metabolic relevance (2). Growth hormone is secreted from the pituitary gland and stimulates IGF-I synthesis and secretion, which in turn inhibits GH secretion via the negative feedback loop (3). The liver is the major contributor to the circulation pool of IGF-I (4), the six IGF-binding proteins (IGFBP-1 to -6) (5) and acid labile subunit (ALS) (6). In the circulation, approximately 99% of the IGF-I pool is bound with high affinity to the IGFBPs that circulate in molar excess of IGF-I, thus explaining that less than 1% circulates as free, unbound IGF-I (7). By serving as carriers of circulating IGF-I, the IGFBPs and ALS prolong IGF-I half-life and modulate tissue access, thereby controlling IGF-I action (5, 8, 9).

**Figure 1:**
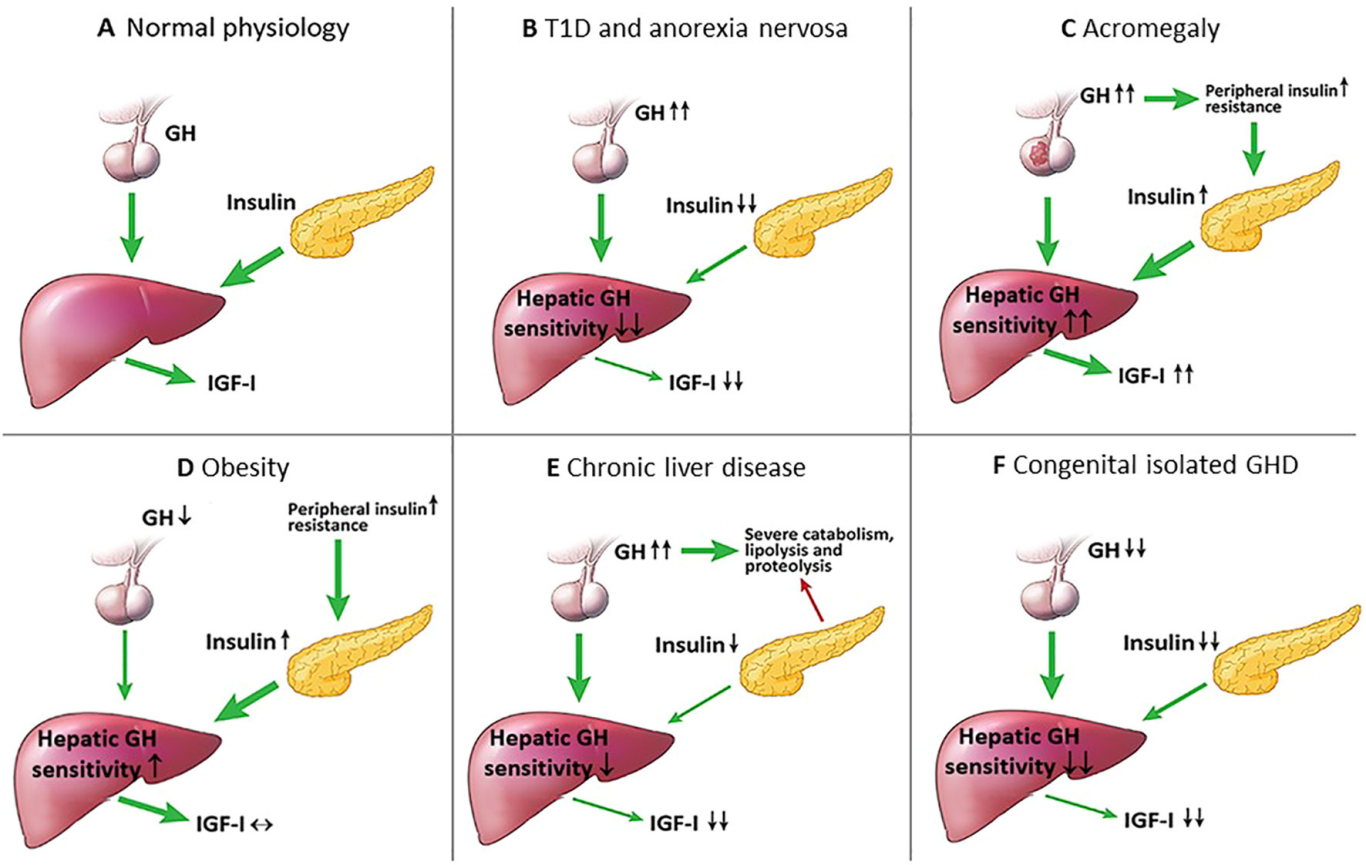
Intra-portal insulin changes in health and several disease states. **(A)** Normal physiological state: intra-portal insulin regulates hepatic GH sensitivity in the generation of IGF-I; **(B)** T1D and anorexia nervosa: intra-portal hypoinsulinemia decreases hepatic GH sensitivity resulting in low IGF-I levels, and due to the lack of negative feedback by IGF-I on the hypothalamus, GH levels increase; **(C)** Acromegaly: GH excess increases insulin resistance causing intra-portal hyperinsulinemia that leads to increased hepatic GH sensitivity, and the combination of increased GH and hepatic GH sensitivity leads to further increase in IGF-I levels; **(D)** Obesity: peripheral insulin resistance causes compensatory intra-portal hyperinsulinemia that increases hepatic GH sensitivity resulting in high normal IGF-I levels and low GH levels; **(E)** Chronic liver disease: the liver fails to produce sufficient IGF-I resulting in high GH levels due to the lack of negative feedback by IGF-I on the hypothalamus that causes a state of catabolism, lipolysis and proteolysis, lipolysis and decreased β-cell insulin secretion; **(F)** Congenital isolated GHD: severe lifelong GHD results in decreased β-cell mass and insulin secretion, and the combination of decreased GH and intra-portal hypoinsulinemia leads to markedly low IGF-I levels.

Insulin, when secreted from pancreatic β-cells, is transported by the portal vein directly to the liver resulting in high hepatocyte exposure. Notably, the liver is a major target organ for the metabolic effects of insulin and exerts a first-pass extraction of up to 85% of insulin delivered by the portal vein (10). Additionally, because of its ability to regulate the hepatic sensitivity to GH, intra-portal insulin delivery has turned out to be an essential co-player for effective GH-induced hepatic IGF-I synthesis (11–15) (**Figure 1A**). Indeed, one may consider the ability of insulin to control the hepatic GH sensitivity to be a normal physiological response to changes in nutrition. During fasting with decreased intra-portal insulin levels, hepatic GH sensitivity decreases leading to decreased serum IGF-I levels despite a compensatory increase in GH secretion. Conversely, overfeeding increases intra-portal insulin levels that enhances hepatic GH sensitivity to a degree where relatively low GH secretion is sufficient to maintain ambient hepatic IGF-I synthesis and secretion, and normal circulating IGF-I levels (7). This GH response to nutritional changes is physiologically appropriate as it leads to increased GH-mediated insulin resistance that prevents hypoglycemia during fasting (16) and decreased GH-mediated insulin resistance during periods of overfeeding (17). However, disease states (e.g., obesity, Cushing’s syndrome, type 1 diabetes [T1D] and anorexia nervosa) and some commonly used drugs [e.g., glucocorticoids and glucagon-like peptide 1 receptor agonists (GLP-1RAs)] that impact pancreatic β-cell insulin secretion can also alter the GH/IGF-I axis. As GH, IGF-I, and insulin continuously modulate each other’s secretion and actions during health and disease, a good understanding of the mechanisms behind these interactions is of clinical importance.

This review summarizes how changes in hepatic exposure to intra-portal insulin in health and disease states modulates the GH/IGF-I axis. We also discuss the relevance of kidney function, another confounder that may alter the GH/IGF-I axis in a manner that can replicate findings seen in acromegaly. Finally, we briefly describe how the GH/IGF-I axis is impacted by liver disease, weight loss, GH misuse for recreational and athletic performance-enhancing, GH-releasing hormone (GHRH) analog use in HIV lipodystrophy, and the alterations of the GH/IGF-I axis in subjects with congenital isolated GH and IGF-I deficiencies.

## Physiology of the GH/IGF-I axis

### Physiology

Pituitary secretion of GH is under the control of GHRH stimulation and somatostatin inhibition, both secreted from the hypothalamus (3). Growth hormone secretion is episodic and pulsatile, with levels varying between peaks and troughs, and very low levels between pulses (18). Growth hormone stimulates IGF-I synthesis and secretion, which feeds back on the pituitary to inhibit GH secretion (19, 20). Insulin-like growth factor-I affects the regulation of GH secretion at the hypothalamus by inhibiting the GHRH gene expression (19) and stimulating somatostatin secretion (20), whereas at the pituitary, IGF-I inhibits spontaneous and GHRH-stimulated GH secretion (3). Unlike the episodic and pulsatile nature of GH secretion, IGF-I is secreted continuously, has a longer half-life and exhibits more stable concentrations in the blood (21). Hence, IGF-I is utilized as a biomarker of GH secretory status, as its levels reflect the 24-hour integrated GH secretion (22–24).

Apart from insulin, there are other hormones that can influence pituitary GH secretion, such as ghrelin, estrogens and androgens. Ghrelin, a gastric peptide with potent GH secretagogue properties, amplifies hypothalamic GHRH secretion and synergizes its pituitary GH-stimulation effects (25). Estrogens stimulate pituitary GH secretion, but inhibit GH action on the liver by suppressing GHR signalling (26). Additionally, estrogens can potentiate ghrelin action (27), while androgens enhance peripheral actions of GH (28). Finally, pituitary GH secretion inversely correlates with intra-abdominal visceral adiposity via mechanisms that depend on free fatty acid (FFA) fluxes (3).

### Insulin

Insulin suppresses GH secretion, but the underlying mechanisms are not well-understood, and are postulated to occur at several different levels. At the level of the pituitary, *in vitro* studies of pituitary cells and *in vivo* findings in mice harvesting a somatotroph-specific knockout of the insulin receptor have demonstrated that insulin directly suppressed pituitary GH secretion independent of the IGF-I receptor (IGF-IR) (29, 30). At the level of the liver, *in vitro* studies using the human hepatoma cell line (HuH7) as a model have shown that insulin stimulates GHR protein synthesis and GHR binding of GH in a dose-dependent manner (31). Similarly, *in vivo* studies based on rodents have shown reduced hepatic GHR expression (32) and GH binding (32, 33) following induction of diabetes, and that insulin treatment facilitated GH binding (33). These findings highlight the key role insulin plays in modulating hepatic responsiveness to GH and support the notion that insulin plays an important part in regulating hepatic GH sensitivity via its ability to directly influence GHR expression and GH binding. However, to the best of our knowledge, this notion is yet to be demonstrated in humans.

**Figure 2:**
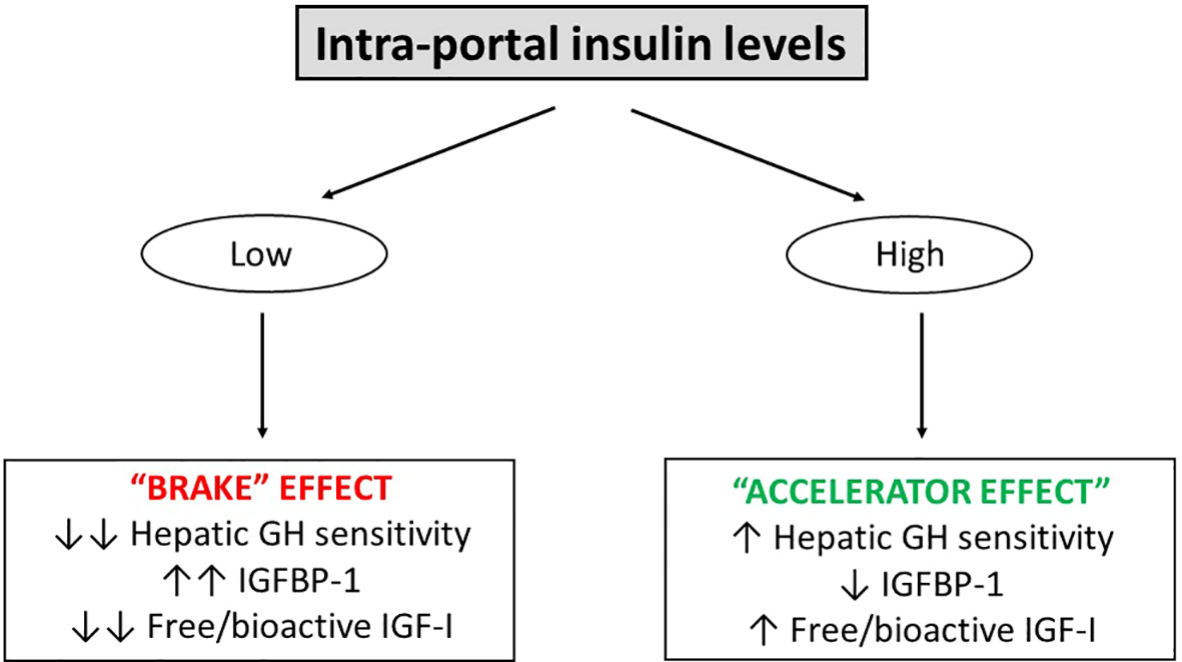
The ability of intra-portal insulin to serve as an “accelerator” and “brake” on hepatic GH sensitivity and free/bioactive IGF-I. In the setting of low intra-portal insulin levels (e.g., overnight fasting and T1D), there is a “brake” effect leading to reduced hepatic GH sensitivity, increased IGFBP-1 and reduced free/bioactive IGF-I. In the setting of high intra-portal insulin levels (e.g., feeding and obesity), an “accelerator” effect takes place leading to increased hepatic GH sensitivity. However, with regards to the “accelerator” and “brake” effects of insulin on serum free/bioactive IGF-I, it appears that the ability of high intra-portal insulin levels to increase (“accelerate”) serum free/bioactive IGF-I activity is less pronounced compared to the ability of low intra-portal insulin levels to decrease (“brake”) serum free/bioactive IGF-I activity. Two arrows indicate a marked effect, one arrow indicates a milder effect.

Furthermore, besides regulating circulating IGF-I levels via its impact on the hepatic GH sensitivity, insulin also inhibits hepatic synthesis and secretion of IGF-binding protein-1 (IGFBP-1); an inhibitor of IGF-I action (7). Thus, the relationship between insulin and IGFBP-I is inverse (34), where IGFBP-1 is low during states of hyperinsulinemia but elevated during states of hypoinsulinemia (35). Because IGFBP-1 levels fluctuate throughout the day (36), this has led to the hypothesis that IGFBP-1 links IGF-I action to nutritional intake. However, present data indicate that IGFBP-1 serves more as a “brake” on IGF-I action by inhibiting anabolic signaling during nutritional depletion than an “accelerator” of IGF-I action during overfeeding. The rationale is that under normal physiological circumstances, the decline in IGFBP-1 from its maximally stimulated overnight fasting level (34) to the level seen after food intake on a molar basis is relatively modest, and only leads to minor increases in serum free IGF-I (7, 37, 38). Another explanation is that the molar concentrations of the other five IGFBPs far exceeds the concentration of IGFBP-1 (39), and as they are not fully saturated, the other five IGFBPs are fully capable of sequestering the increase in free IGF-I secondary to the reductions in circulating IGFBP-1. However, when IGFBP-1 levels are chronically lowered during prolonged intra-portal hyperinsulinemia (e.g., obesity, Cushing’s syndrome and glucocorticoid therapy), the “accelerator” effect of IGFBP-1 is at play to increase serum free IGF-I (35, 40). Conversely, during prolonged intra-portal hypoinsulinemia (e.g., malnutrition, anorexia nervosa, and T1D), IGFBP-1 levels increase several-fold (7), leading to the increased complex formation between IGF-I and IGFBP-1 and clear reductions in free IGF-I (“brake” effect) (**Figure 2**).

There is also now ample evidence demonstrating that insulin reaching the liver via the portal system plays an important role in making the liver more GH sensitive to IGF-I generation (23, 41, 42). Disease states of GH excess (e.g., acromegaly) and GH deficiency (GHD) (e.g., congenital isolated GHD) are characterized by increased and decreased GH and IGF-I levels, where the GH/IGF-I relationship is reflected by a “primary association” (high GH and high IGF-I for acromegaly and low GH and low IGF-I for GHD). However, certain disease states and drug therapies can influence intra-portal insulin levels and invariably alter the relationship between GH and IGF-I. For instance, when intra-portal insulin levels are increased [e.g., obesity, Cushing’s syndrome or due to treatment with glucocorticoids and glucagon-like peptide-1 receptor agonists (GLP-1RA)] or decreased (e.g., anorexia nervosa and T1D) in disease states, these secondary changes (“secondary association”) alters hepatic GH sensitivity resulting in discordant GH and IGF-I levels (high GH/low IGF-I levels and low GH/high IGF-I levels, respectively).

## Effects of disease states on intra-portal insulin levels and changes in the GH/IGF-I axis

### High GH and low IGF-I

**Figure 3:**
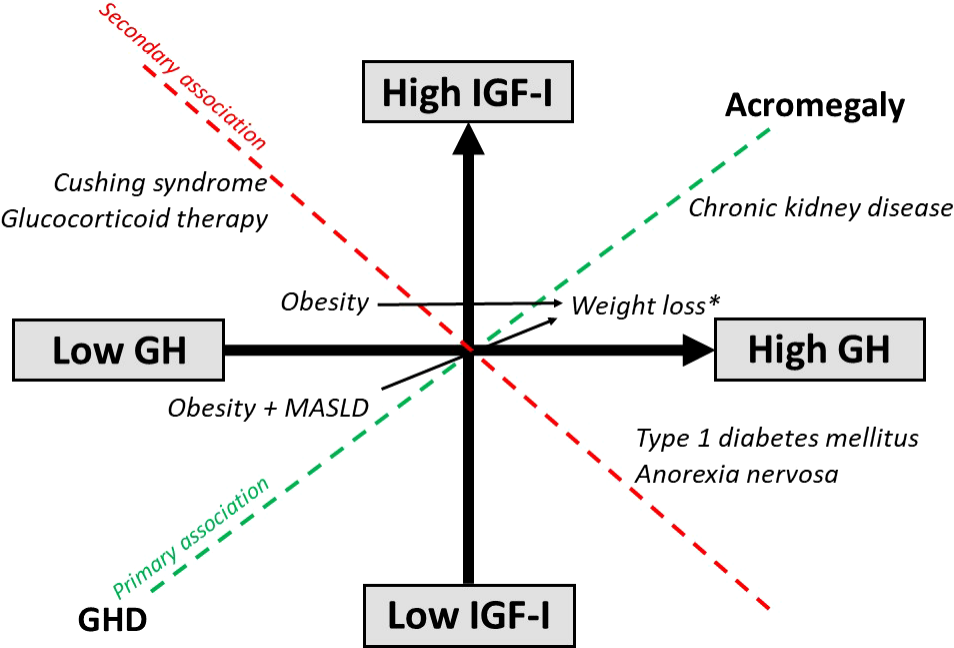
Effects of disease states and weight loss on the GH/IGF-I axis. *Weight loss induced by diet, glucagon-like peptide 1 receptor agonist therapy and surgery.

Type 1 diabetes is an autoimmune disease characterized by pancreatic β-cell destruction leading to intra-portal hypoinsulinemia. This disease state provides an ideal model to illustrate the pivotal role of intra-portal insulin as the key regulator of the GH/IGF-I axis. Patients with uncontrolled T1D have high GH, low total and free IGF-I levels (**Table 1**; **Figures 1B**, **3**), and elevated IGFBP-1 levels (2, 43, 44). Following subcutaneous insulin treatment, IGFBP-1 decreases while serum total and free IGF-I increases (43, 44). Notably, changes in IGFBP-1 and free IGF-I are inversely correlated (44), thereby emphasizing the “brake” effect of IGFBP-1 on IGF-I action. The “brake” effect of IGFBP-1 is further substantiated by Attia et al. (45), who paused continuous subcutaneous insulin infusion (CSII) for 8 hours in patients with T1D, and found that IGFBP- 1 levels increased by 6-fold, whereas total and free IGF-I levels decreased. Importantly, intra-portal hypoinsulinization in T1D and its dysregulatory effect on GH/IGF-I axis (**Table 1**; **Figures 1B**, **3**) is not fully ameliorated by conventional subcutaneous insulin administration, most likely because subcutaneous administration of insulin cannot fully compensate for the normal portal delivery of insulin to the liver. This notion is supported by several clinical studies of patients with T1D. Shishko et al. (13) reported that intra-portal insulin infusion (IPII) was superior to CSII with regards to suppression of IGFBP-1, and normalization of IGF-I and 24-hour GH profiles. These findings were corroborated by Hannaire-Broutin et al. (11), who switched patients from CSII to intra-peritoneal insulin infusion and reported improvements in serum levels of total IGF-I and GH-binding protein (GHBP); the latter serving as a proxy of hepatic GHR density. These findings indicate that intra-peritoneal insulin delivery stimulates hepatic GH sensitivity and improves hepatic IGF-I generation. Hedman et al. (12) demonstrated that intra-peritoneal insulin increased total and bioactive IGF-I, and decreased IGFBP-1 levels. In a study by Frystyk et al. (46) comparing T1D patients, who received combined kidney and pancreas transplantation, one group received a pancreatic graft allowing portal delivery of insulin, while the other, a graft with systemic pancreas drainage. Despite obtaining similar hemoglobin A1c levels, serum total and free IGF-I were elevated and IGFBP-1 were suppressed in patients receiving a pancreas with portal drainage. In another study, when comparing T1D C-peptide positive pre-pubertal children and adults *vs* their C-peptide negative counterparts, patients with a residual β-cell function had higher IGF-I levels despite similar glycemic control (47, 48). In alignment with these findings, the existence of residual pancreatic β-cell function is of fundamental metabolic importance in T1D. Collectively, these findings indicate that glycemic control does not solely influence the GH/IGF-I axis in T1D and that small changes in hepatic insulinization can exert more meaningful systemic effects (49). Abnormalities in the GH/IGF-I axis have also been observed in patients with type 2 diabetes (T2D) (50). Studies have shown that exogenous administration of recombinant human IGF-I (51, 52) and IGF-I/IGFBP-3 complex (53–55) improved insulin sensitivity and decreased insulin requirements in both T1D and T2D patients. Nevertheless, the clinical implications of high GH and low IGF-I on long-term metabolic and vascular outcomes in patients with T2D remain unclear.

**Table 1:** Summary of the changes in intra-portal insulin levels in different disease states, after weight loss and after drug therapy on the GH/IGF-I axis.

Disease states	GH	IGF-I	Intra-portal insulin	Hepatic GH sensitivity
Acromegaly	↑↑	↑↑	↑	↑
Chronic kidney disease*	↔/↑	↔/↑	↔/↑	↔/↓
Congenital growth hormone deficiency	↓↓	↓↓	↓	↓
Type 1 diabetes mellitus	↑↑	↓↓	↓↓	↓
Anorexia nervosa	↑↑	↓↓	↓↓	↓
Inflammatory diseases	↑	↓	↔/↑	↓
Obesity^#^	↓	↔	↑	↑
MASLD	↔/↓	↓	↑	↔/↓
Obesity followed by weight loss^	↑	↔	↓	↓
Chronic liver disease and catabolic states	↑↑	↓↓	↓	↓
Cushing’s syndrome	↓	↑	↑	↑
Glucocorticoid therapy	↓	↑	↑	↑
Growth hormone misuse for recreational and athletic performance-enhancing^¶^	↑	↑	↑	↑
Growth hormone-releasing hormone analogue use in HIV lipodystrophy	↑	↑	↔	↔

↑, increased; ↓, decreased; ↔, unchanged.

*Dependent on the extent of renal dysfunction and age of the patient.

^#^GH levels tends to be low in obesity and negatively correlates with body mass index.

^Weight loss can be induced by diet, glucagon-like peptide 1 receptor agonist therapy and surgery.

^¶^Supraphysiological doses of GH are often used.

MASLD; metabolic dysfunction-associated steatotic liver disease.

Anorexia nervosa is a condition of severe undernutrition characterized by GH resistance secondary to chronic nutritional deprivation with resultant high GH and low serum levels of free, bioactive and total IGF-I (56). Patients with the lowest BMI and fat mass tended to have the highest serum GH levels (57, 58). The low IGF-I levels is caused by nutritionally acquired hepatic GH resistance from intra-portal hypoinsulinemia (**Table 1**; **Figures 1B**, **3**), resulting in down-regulation of the hepatic GHR expression, as inferred from reductions in circulating levels of GHBP (59), and decreased GH binding (60). The state of hepatic GH resistance in anorexia nervosa is further corroborated by the lack of increase in IGF-I following administration of supraphysiological doses of exogenous GH in women with anorexia nervosa (61), and only refeeding appears to able to restore IGF-I levels (56, 59, 62). Therefore, in states of chronic under-nutrition, hepatic GH resistance is an adaptive response, as high GH levels are necessary to maintain euglycemia, while low IGF-I levels conserves energy during periods of nutritional deprivation. Other non-insulin mechanisms of hepatic GH resistance in anorexia nervosa have also been described, including fibroblast growth factor-21, sirtuin 1, triiodothyronine, leptin and testosterone (63).

In the setting of chronic inflammatory disease states (e.g., Crohn’s disease or juvenile chronic arthritis), these diseases can also disturb the physiological synergy of the GH/IGF-I axis. The presence of insulin resistance and elevated pro-inflammatory cytokines (e.g., tumor necrosis factor-α) induce hepatic GH resistance (64), causing increases in GH and decreases in IGF-I levels (**Table 1**). However, the GH/IGF-I axis in these disease states generally resets itself following the resolution of the inflammatory trigger (65).

### High GH and high IGF-I

One of the most well-described primary GH disorders of high GH and high IGF-I is acromegaly (66). In active disease, cellular responses elicited by high GH levels can overwhelm the intracellular mechanisms thus attenuating GH signaling (67). Additionally, the GH stimulatory effect on the liver to increase IGF-I secretion is accentuated by the direct GH effects in antagonizing insulin action and suppressing the anti-lipolytic actions of insulin (68). This leads to peripheral and hepatic insulin resistance and compensatory intra-portal hyperinsulinemia, which results in increased hepatic GH sensitivity that aggravates the condition further (**Table 1**; **Figures 1C**, **3**). Supportive of this notion, a proof-of-concept study performed by Coopmans et al. (69) demonstrated that 14-day exposure to a eucaloric very-low-carbohydrate ketogenic diet as adjuvant treatment to first-generation somatostatin receptor ligands decreased IGF-I without increasing GH levels in acromegaly patients. Although measures to study insulin secretion were not performed, this study suggested that even in the context of uncontrolled acromegaly and insulin resistance, insulin still plays a key role in the regulation of circulating IGF-I levels. The role of insulin in controlling hepatic IGF-I was further illustrated in a study of moderately obese subjects undergoing caloric restriction and receiving GH. In this study, two groups of subjects received a severely energy-restricted diet. In one group, most of the calories were supplied as carbohydrate and in the other, as lipid. The subjects receiving most of their energy as fat had a lesser IGF-I response to GH, whereas subjects receiving the same caloric intake as carbohydrate demonstrated greater increases in IGF-I to GH stimulation (70, 71). When serum insulin or 24-hour urinary C-peptide were measured, those receiving carbohydrate had higher serum insulin levels and 24-hour urinary C-peptide. It is also possible that in active acromegaly, chronic exposure to elevated IGF-I levels resets IGF-IR responsiveness via the induction of IGF-IR resistance (70), as it has been demonstrated that prolonged stimulation with IGF-I induces functional IGF-IR resistance *in vitro* through the negative feedback loop (72). Thus, in the setting of IGF-IR resistance in the pituitary and hypothalamus of acromegaly patients, the negative feedback loop by IGF-I may be defective resulting in further IGF-I elevations.

**Figure 4:**
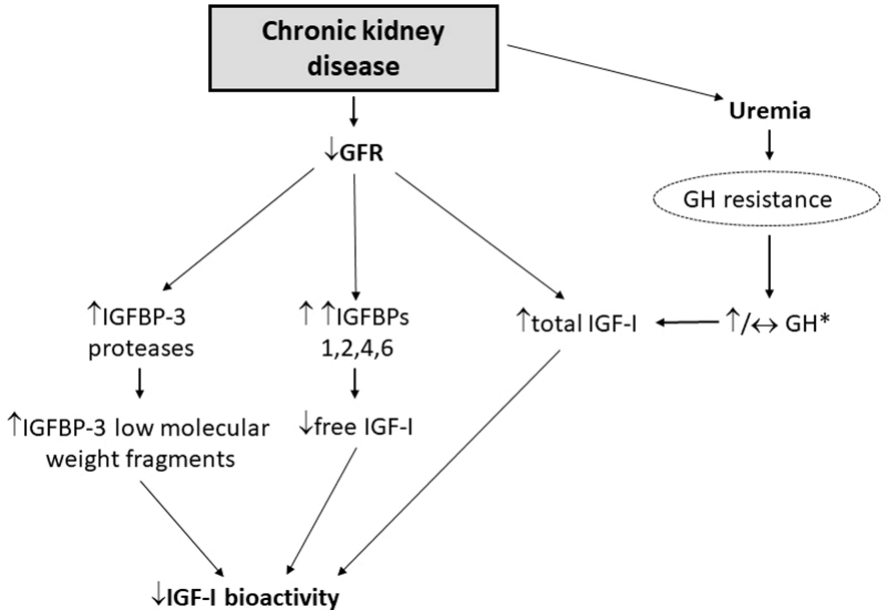
Deranged GH/IGF-I axis in patients with CKD [adapted from Kamenicky et al. (77)]. *Random fasting GH levels can either be increased or normal, depending on the extent of renal dysfunction manifested by the degree of uremia and age of the patient.

Chronic kidney disease (CKD) is a condition that mimics some components of the biochemical picture of acromegaly with high serum GH and total IGF-I levels. The kidneys contribute to GH degradation and as a result, GH half-life is increased in patients with CKD due to decreased renal clearance (73) (**Figure 4**). Some investigators have suggested that the uremia of CKD is associated with decreased GH signalling contributing to the state of hepatic GH resistance (74) (**Figure 4**). Hence, depending on the extent of renal dysfunction manifested by the degree of uremia and age of the patient (75, 76), random fasting GH levels can either be increased or normal. Impaired kidney function is also associated with increased IGFBP levels (e.g., IGFBP-1, -2, -3, -4, and -6) (75, 77) and IGFBP-3 protease activity, which subsequently increases low-molecular-weight IGFBP-3 fragments (78), most likely due to a compromised renal IGFBP-clearance (**Figure 4**). The increase in IGFBP-1 and -2 leads to secondary increases in circulating IGF-I binding capacity that results in high total IGF-I, but decreased IGF-I bioactivity (77, 78) (**Figure 4**). It is also noteworthy that due to the excess circulating IGFBPs in end-stage renal failure patients on hemodialysis, GH is only approximately 50% efficient in increasing bioactive IGF-I compared with healthy subjects (79). Furthermore, peripheral insulin resistance triggered by uremia, metabolic acidosis and low-grade inflammation is common in CKD (80), leading to intra-portal hyperinsulinemia, but does not necessarily increase hepatic sensitivity to GH because of the reduced hepatic expression of IGF-I and GHR mRNA (81). Thus, high total IGF-I levels in CKD is inadequate in suppressing the increased GH levels because of the lower IGF-I bioactivity resulting in the lack of IGF-I feedback on pituitary GH secretion (75, 77) (**Table 1**; **Figure 3**).

### Low GH and low IGF-I

Like acromegaly, GHD can be considered as a primary GH disorder, where changes on the GH/IGF-I axis are reflected by a primary association with low GH and low IGF-I (**Table 1**; **Figure 3**). Patients with GHD can present as either childhood-onset (CO-GHD) or adult-onset (AO-GHD). Furthermore, GHD may occur as isolated GHD or with multiple pituitary hormone deficiencies. The most common cause of CO-GHD is idiopathic and can be isolated or associated with other pituitary hormone deficiencies, while AO-GHD is frequently acquired from hypothalamic-pituitary tumors and/or secondary to their treatment (82). Because IGF-I exerts anti-inflammatory actions and is important for peripheral glucose uptake, metabolic disturbances in these patients can, in part, be explained by the low IGF-I levels (83). Furthermore, due to deficient GH secretion, the ability of GH to antagonize the anti-lipolytic effect of insulin is reduced, leading to accumulation of visceral adipose tissue (VAT). These effects promote insulin resistance and intra-portal hyperinsulinemia that increases hepatic GH sensitivity to maintain some degree of IGF-I secretion. Conversely, CO-GHD patients tend to be less insulin-resistant than AO-GHD patients (84), and their IGF-I levels are generally lower than their AO-GHD counterparts due to relatively lower intra-portal insulin levels. Thus, insulin resistance leading to elevated portal insulin levels may explain, at least partially, the overlap in individual IGF-I levels between older GH-deficient subjects and GH-sufficient subjects (85, 86). This is clinically relevant, as IGF-I is generally less reliable of a biomarker for screening adults above 60 years with GHD and is a better screening and diagnostic biomarker in GHD in subjects with disease onset in childhood or early adulthood (86).

### Low GH and normal/high IGF-I

Obesity is a common yet serious condition associated with blunted spontaneous and stimulated GH secretion (87, 88), i.e., a state of relative GHD. However, serum total and bioactive IGF-I are relatively preserved despite reductions in GH levels (89) (**Table 1**; **Figures 1D**, **3**). Previous studies have found that GH levels in patients with obesity was decreased nearing the lowest levels of normal range in men and women (90), with levels in those with morbid obesity comparable to adults with GHD (91). As weight loss restores GH levels (92–94), this indicates that the blunted GH secretion is an effect rather than a cause of obesity. This paradox may be explained by the increased insulin resistance and the compensatory intra-portal hyperinsulinemia causing increased hepatic GH sensitivity that increases serum IGF-I and blunts pituitary GH secretion via the negative feedback loop. The increased hepatic exposure to insulin may also explain why obese subjects following exogenous GH administration respond better with higher increases of IGF-I than lean subjects; the main concept behind the IGF-I generation test (95). Additionally, elevations in FFAs in obesity contributes to GH suppression, as experimental lowering of FFA levels reverses obesity-associated impairment of GH secretion (96). Collectively, it may be hypothesized that obesity induces feedback inhibition of pituitary GH secretion by increasing non-fasting serum FFA levels and the associated insulin resistance that causes compensatory intra-portal hyperinsulinemia and IGFBP-1 suppression, with the net result being low GH and normal IGF-I levels. Whether this unique pattern worsens the obese state or constitutes a favorable adaptation remains unclear, and the physiological and pathophysiological implications of the altered GH/IGF-I axis in obesity remain uncertain with GH neither being the cause nor the solution to reverse obesity. Notably, in mice studies, GH secretagogue receptor suppression in adipose tissues protects against obesity and insulin resistance (97), whereas IGF-I increases skeletal muscle insulin sensitivity (98). Thus, it is possible that the combination of low GH and normal IGF-I serves as a physiological mechanism aimed at preserving the GH/IGF-I anabolic effects and minimizing the insulin-antagonistic effects of GH without compromising the positive IGF-I metabolic effects.

The hypothalamic-pituitary-adrenal and the GH/IGF-I axis are also closely associated, as cortisol is required for physiological GH secretion. In states of hypercortisolemia (e.g., Cushing’s syndrome), decreased GH secretion with normal to elevated serum IGF-I levels have been reported (99, 100) (**Table 1**; **Figure 3**). English et al. (101) assessed serum IGF-I levels in patients with Cushing’s disease and any changes following post-operative remission compared to matched controls being investigated for suspected pituitary dysfunction (e.g., nonfunctioning pituitary adenomas). These investigators found that untreated Cushing’s disease patients had low GH and high IGF-I levels without clinical features of acromegaly, and after surgery with disease remission, serum IGF-I levels normalized. Magiakou et al. (100) demonstrated decreased 24-hour GH secretion despite the mean IGF-I SDS being +1.0 in patients with Cushing’s disease, and Bang et al. (102) reported elevated IGF-I levels in 40% of patients with pituitary and adrenal Cushing’s syndrome. Similar to obese patients, patients with Cushing’s syndrome also responded with supranormal IGF-I levels following administration of exogenous GH (95, 103). An interesting observation that deserves further exploration is the inherent ability of intra-portal insulin to regulate hepatic GH sensitivity that remains preserved despite prevailing changes in peripheral insulin resistance. This observation has been reported in earlier studies in obese and hyperinsulinemic subjects, and in patients with Cushing’s syndrome, in whom hepatic sensitivity to GH is increased when compared to lean subjects (95, 103). However, the exact molecular mechanisms responsible for this preserved effect remains unclear.

**Figure 5:**
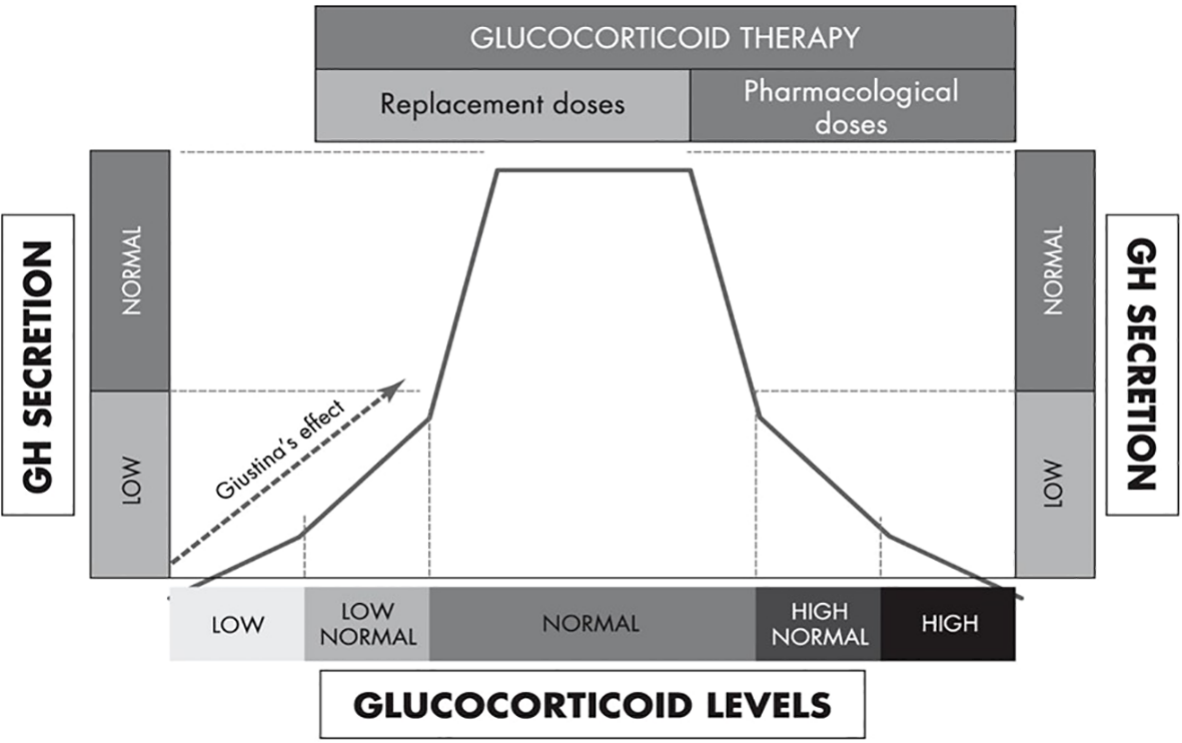
An integrated model of the biphasic, dose-dependent effect of glucocorticoids on GH secretion [adapted from Mazziotti et al. (104)].

Further evidence of hypercortisolemia decreasing GH and increasing IGF-I comes from data of exogenous glucocorticoid therapy in humans. Excess glucocorticoid exposure suppresses GH secretion mainly through the increase in hypothalamic somatostatin tone, whereas low cortisol levels causes relative GHD that is reversible by glucocorticoid therapy (“Giustina’s effect”) (**Figure 5**) (104). Other mechanisms include increased insulin resistance induced by high-dose glucocorticoids that increases intra-portal insulin, decreases IGFBP-1 that increases free IGF-I, and direct glucocorticoid effects in increasing hepatic IGF-I synthesis and decreasing hepatic insulin clearance (105). Prummel et al. (106) demonstrated that prednisone therapy rapidly increased serum IGF-I levels; however, these levels promptly returned to baseline after prednisone discontinuation followed by suppressed GH levels. Ramshanker et al. (107) demonstrated that even though prednisolone increased serum total and bioactive IGF-I, post-IGF-IR signaling was inhibited indicating that prednisolone-induced increases in serum IGF-I levels is not accompanied by increased IGF-I action. This observation aligns with the findings by Unterman et al. (108), who utilized a porcine cartilage bioassay and demonstrated that glucocorticoid treatment in children decreased IGF-I activity. Finally, there are also data showing that even though serum IGF-I is acutely increased following prednisolone, this is not the case in lymph-like tissue fluid (107), suggesting that glucocorticoids exert distinct, compartment-specific effects on IGF-I action. Collectively, these findings raise the possibility that the mechanism of glucocorticoids increasing serum IGF-I levels is by directly inducing post-IGF-IR signaling resistance.

## Effects of weight loss

Short-term fasting is an important stimulator of GH secretion (109, 110) (**Table 1**; **Figure 3**). The increase in GH secretion precedes the reduction in IGF-I levels that is only evident by the third day of fasting, thereby ruling out the increase in GH during fasting is related to reductions in total IGF-I. However, when measuring free and bioactive IGF-I, it appears that serum levels decreased concomitantly with increases in IGFBP-1 and GH. Thus, during short-term fasting, changes in free and bioactive IGF-I rather than total IGF-I appear to be at least in part responsible for the increased GH secretion (35). Conversely, despite weight loss and concomitant improvements in insulin sensitivity, neither low caloric diets (111, 112), intermittent fasting (113), nor long-term calorie restriction (114) exerts any consistent inhibitory impact on serum IGF-I levels. One explanation for this may be that as long as the intake of protein is sufficient, serum IGF-I levels will remain relatively stable (115), i.e., the intake of calories *per se* does not alter serum IGF-I levels. In the last two decades, bariatric surgery has been highly successful in treating obesity (116), and changes in the GH/IGF-I axis has been extensively studied in this context. Changes in GH appear to be consistent, with several studies reporting increased GH levels following surgery (92–94), whereas the response of total IGF-I to bariatric surgery is more variable (117). We have reported that serum total IGF-I decreased 1 week and 3 months after bariatric surgery, whereas serum bioactive IGF-I remained unchanged during that period, and after 12 months, both variables returned to pre-surgery levels (117). The variability in total IGF-I may be due, in part, to major changes in selective IGFBPs during the post-bariatric surgery period, i.e. the proteolysis of IGFBP-3 (118). Another possible explanation may be the presence or absence of metabolic dysfunction-associated steatotic liver disease (MASLD), which can directly influence hepatic IGF-I generation.

## Effects of MASLD, chronic liver disease and GLP-1RA therapy

Metabolic dysfunction-associated steatotic liver disease (MASLD) is highly prevalent in obese individuals with insulin resistance (119, 120). However, in contrast to simple obesity, where GH is low and IGF-I within normal range (7, 89), subjects with MASLD demonstrate variable changes in the GH/IGF-I axis depending on disease severity (121–123) (**Table 1**). In simple steatosis and early metabolic dysfunction-associated steatohepatitis (MASH), many of the body composition and metabolic features predisposing to MASLD/MASH contribute to decreased GH and IGF-I levels. With increasing hepatic dysfunction, the GH signaling cascade and hepatocyte IGF-I synthesis becomes impaired (124). When cirrhosis develops, a state of hepatic GH resistance ensues resulting in high GH and low IGF-I levels (125) (**Figure 1E**). Cianfarani et al. (126) and Dichtel et al. (127) have shown that circulating IGF-I levels are related to the histological stages of MASLD. In a recent study aimed at investigating IGF-IR and GHR physiology in patients with MASLD, Osganian et al. (128) demonstrated that GHR and IGF-IR expression hepatocytes were comparable across MASLD severity. However, the IGF-I gene expression decreased with increasing severity of MASLD, suggesting that reductions of hepatic IGF-I production in patients with MASLD may be related to the severity of the damaged hepatocytes. It is also possible that insulin resistance is the link between low GH levels and MASLD, as insulin directly suppresses pituitary GH secretion (29, 30). Furthermore, inflammation is a characteristic feature of MASLD (129). Thus, MASLD patients contrast to patients that have progressed to liver cirrhosis, as these patients are more susceptible to developing sarcopenia, a chronic catabolic state with increased energy expenditure, decreased appetite, low protein synthesis, and the development of ascites or portosystemic shunts that contribute to muscle atrophy (130). In these patients, when total hepatocyte mass decreases, IGF-I synthesis and glycogen stores decrease (131) resulting in low glucose and intra-portal insulin levels, and a state of hepatic GH resistance ensues. This inevitably results in low IGF-I and high GH that may stray into the acromegaly range (132, 133) and this catabolic state is comparable to prolonged fasting and malnutrition states (**Figure 1E**). Therefore, IGF-I deprivation contributes to the progressive malnutrition in chronic liver disease patients, increasing the vulnerability of the liver to an inflammatory and oxidative microenvironment that increases the risk of cirrhosis development.

Obesity is currently a growing public health concern (134) and as weight loss improves many obesity-related complications (135), there is a great deal of interest in developing weight-loss drugs. One of the most commonly studied weight-loss drugs, GLP-1RAs, were initially developed to treat T2D by reducing postprandial glucose excursions, augmenting glucose-dependent insulin release, inhibiting glucagon secretion and delaying gastric emptying (136, 137). However, GLP-1RAs are also effective in promoting weight loss by decreasing appetite and hunger, and increasing satiety (91). Heinla et al. (138) demonstrated that administration of a single dose short-acting GLP-1RA exenatide increased GH levels, whereas administration of long-acting GLP-1RA liraglutide daily for 21 days elicited increased GH levels with no changes in IGF-I levels in healthy subjects. Acute GLP-1RA infusion has been shown to decrease IGFBP-3 levels in humans, and this effect may explain the observed decrease in total IGF-1 levels (139). Alternatively, changes in GH and IGF-I levels may secondarily reflect the decrease in body weight following liraglutide treatment. Taken together these results provide further support that GLP-1RAs can alter the functioning of the GH/IGF-I axis.

## Effects of GH misuse for recreational and athletic performance and GHRH analogue use in HIV lipodystrophy

The notion that GH enhances physical function due to its anabolic and lipolytic properties, albeit unproven, and difficulty of detection is what drives its misuse for recreational and athletic performance enhancement to confer a competitive advantage. Athletic performance depends on muscle strength and the energy required to increase muscle function. In recreational athletes, supraphysiological GH doses are often used either alone or in combination with other doping substances (140), and studies have shown that GH improves anaerobic sprint capacity, but not strength and endurance (141), and increases GH, IGF-I and fasting insulin levels (142, 143) mimicking those seen in acromegaly patients (**Table 1**).

Given the evidence that a pulsatile pattern of GH secretion is important for many of its physiological actions, a strategy to augment physiological pulsatile GH secretion without inducing the side-effects of supraphysiological GH doses has been studied in HIV lipodystrophy with excess VAT and decreased endogenous GH secretion (144, 145). Tesamorelin, a synthetic GHRH analog, that stimulates the synthesis and release of endogenous GH, is the first and, so far, only treatment indicated for the reduction of excess abdominal fat in HIV lipodystrophy (144). In a systematic review of 10 clinical trials in patients with HIV lipodystrophy, tesamorelin was effective in reducing VAT and increasing lean body mass and did not alter subcutaneous adipose tissue (146), and increased GH and IGF-I while insulin and glucose levels are unchanged indicating preservation of insulin sensitivity (147–150) (**Table 1**). However, this effect is transient and reversed upon discontinuation of therapy, and the long-term risk-benefit analysis of its administration is still unclear.

## Learning lessons from the Ecuadorian Laron and Brazilian Itabaianinha cohorts

The state of severe GH excess and GHD exists on a spectrum from complete to partial. In states of mild GH excess and mild GHD with varying intra-portal insulin levels, the effects of insulin on the GH/IGF-I axis may not be too apparent. However, conditions caused by genetic mutations that affect growth are associated with a wide range of phenotypic abnormalities that may affect glucose handling, primarily due to diminished pancreatic β-cell mass and subsequent intra-portal hypoinsulinemia. The Ecuadorian Laron and Brazilian Itabaianinha cohorts with congenital mutations in the GH and GHRH receptors, respectively, both provide a unique and arguably the purest examples to evaluate the effects of chronic severe IGF-I and both severe GH and IGF-I deficiency, respectively, with severe hypoinsulinemia being the common denominator in both cohorts (151, 152) (**Table 1**; **Figures 1F**, **3**). However, despite low intra-portal insulin levels, low IGF-I and increased fat mass and decreased lean body mass, their glucose tolerance and insulin sensitivity remains unaffected and in fact, is comparable to normal controls (151, 152). These cohorts serve as compelling examples of the lack of direct GH counter-regulatory effects on pancreatic β-cell insulin secretion, thus implying that circulating insulin exerts its glucose-lowering effects more efficiently in these subjects compared with healthy controls.

## Conclusions

Changes in intra-portal insulin levels play an intricate yet permissive role in the regulation of the GH/IGF-I axis by either stimulating or suppressing hepatic GH sensitivity and hepatic IGF-I generation, probably via an insulin-mediated regulation of the hepatic GHR expression. Disease states that affect intra-portal insulin levels by increasing insulin resistance causing compensatory intra-portal hyperinsulinemia or by decreasing pancreatic β-cell insulin secretion causing intra-portal hypoinsulinemia results in changes to hepatic IGF-I synthesis and secretion, and consequently changes in GH levels via the negative feedback loop. Hence, changes in intra-portal insulin levels need to be taken into consideration to correctly interpret the alterations in the GH/IGF-I axis in healthy and disease states as this helps the clinician in formulating the decision-making process of when and how to perform appropriate diagnostic work-up and whether to offer treatment to improve any accompanying metabolic consequences.

For example, if a patient presents with elevated GH and IGF-I, it is important to rule out underlying impairment in renal function as elevated serum GH and IGF-I levels may also be present in patients with CKD, and not diagnose the patient as having acromegaly. For a patient with untreated Cushing’s disease or on high-dose glucocorticoid therapy, an elevated serum IGF-I may be observed, but levels decrease following remission or cessation of glucocorticoid therapy, respectively; hence mildly elevated IGF-I levels in these patients does not imply pathological GH excess. As for patients with GHD and concurrent diseases with intra-portal hypoinsulinemia (e.g., T1D and inflammatory diseases), higher GH doses are required to normalize IGF-I levels, which has cost implications. Conversely, in patients with anorexia nervosa, even supra-physiologic GH doses (mean maximum daily dose: 1.4 mg/day) may not be sufficient to normalize IGF-I levels (61) indicating the important role of hepatic GH sensitivity in successful IGF-I generation. In patients with obesity, there is no evidence for metabolic benefits of GH therapy in the absence of true GHD (153); instead, insulin resistance may worsen. Growth hormone secretion tends to return to normal after weight loss indicating that low GH is a consequence, not a cause, of central obesity (94), and therefore GH therapy should not be used to promote weight loss. As for individuals misusing GH for recreational and athletic performance-enhancing purposes, GH, IGF-I and insulin levels are increased suggesting that insulin sensitivity is worsened and “iatrogenic acromegaly” is induced, especially if used long-term. Conversely, for individuals with HIV lipodystrophy treated with tesamorelin, GH and IGF-I levels are increased but insulin sensitivity remains preserved.

While IGF-I remains the biomarker that best reflects endogenous GH secretion, when there is a discordancy with GH and IGF-I levels, it is important for the clinician to be mindful of the associated changes in intra-portal insulin delivery to the liver when interpreting these results in the context of health and when deciding treatment regimens in disease states. Finally, some commonly used drugs (e.g., glucocorticoids and GLP-1RAs) can also affect intra-portal insulin levels that modulate the GH/IGF-I axis either by increasing or decreasing hepatic GH sensitivity, as reflected by the discordant GH and IGF-I levels.
